# Supplementary material for: Biomass smoke inhalation promotes neuroinflammatory and metabolomic temporal changes in the hippocampus of female mice
Source: J Neuroinflammation. 2023 Aug 22;20:192. doi: 10.1186/s12974-023-02874-y (PMC10464132; doi:10.1186/s12974-023-02874-y)
Supplement: Supplementary file 1 — Additional file 1: Table S1. List of all metabolites affected from limma-based linear modelling. Fig. S1. Neuroinflammatory and Metabolomic Temporal Dynamics. [file 12974_2023_2874_MOESM1_ESM.docx]

Additional file 1

Additional file 1: **Table S1. List of all metabolites affected from limma-based linear modeling.**

| Metabolite | logFC | AveExpr | t | P.Value | adj.P.Val |
| --- | --- | --- | --- | --- | --- |
| 3-(Sulfooxy)benzenepropanoic acid | 431700 | 409420 | 8.6144 | 1.61E-10 | 1.68E-07 |
| Isoprene | -227480 | 264780 | -4.4437 | 7.25E-05 | 0.037657 |
| Octanal | -597260 | 731000 | -4.2283 | 0.00014 | 0.048429 |
| Dinitrosopentamethylenetetramine | -361490 | 1575000 | -4.0344 | 0.000251 | 0.052072 |
| Triethylene glycol monomethyl ether | -361490 | 1575000 | -4.0344 | 0.000251 | 0.052072 |
| 1-(1Z-octadecenyl)-2-linoleoyl-sn-glycero-3-phosphoethanolamine | -108120 | 300230 | -3.8837 | 0.000392 | 0.067884 |
| 6-(4-methoxyphenyl)pyrimidine-2,4-diamine | -2892900 | 8481100 | -3.6079 | 0.000876 | 0.12995 |
| Sebacic acid | -349450 | 1245600 | -3.3623 | 0.001756 | 0.20561 |
| N-{[2-(2-thienyl)-1,3-thiazol-4-yl]methyl}benzamide | -208120 | 535050 | -3.3502 | 0.001817 | 0.20561 |
| asn-arg | -100040 | 218480 | -3.3137 | 0.002011 | 0.20561 |
| Traumatic Acid | -104210 | 568130 | -3.2525 | 0.002381 | 0.20561 |
| Isophorone | -96086 | 486920 | -3.229 | 0.00254 | 0.20561 |
| 2-amino-N-(1,3,5-trimethyl-1H-pyrazol-4-yl)benzamide | -263940 | 519600 | -3.2244 | 0.002573 | 0.20561 |
| 635994 | -111840 | 563300 | -3.1449 | 0.003196 | 0.23699 |
| 2740 | -61513 | 377310 | -3.1196 | 0.003422 | 0.23699 |
| Diaveridine | -1.1E+07 | 29389000 | -3.0883 | 0.003723 | 0.24178 |
| L-decyline | -57099 | 259930 | -3.0017 | 0.004692 | 0.26711 |
| methylguanidine | -57099 | 259930 | -3.0017 | 0.004692 | 0.26711 |
| acetic acid geranyl ester | -411070 | 2441400 | -2.9793 | 0.004979 | 0.26711 |
| Naphthalene | -74252 | 369850 | -2.9569 | 0.005282 | 0.26711 |
| Leu-Val | 351240 | 3011700 | 2.9486 | 0.005399 | 0.26711 |
| Perchloric acid | -114040 | 585760 | -2.9233 | 0.00577 | 0.27248 |
| ecabet | -1634200 | 6179100 | -2.8861 | 0.00636 | 0.2749 |
| 3-Isopropenylpentanedioic acid | -790710 | 6759700 | -2.8848 | 0.006381 | 0.2749 |
| beta-D-glucosyl crocetin | -58611 | 89794 | -2.8519 | 0.00695 | 0.2749 |
| Pantothenic acid | 1192300 | 7675600 | 2.8504 | 0.006977 | 0.2749 |
| 2908 | -1984700 | 6342200 | -2.832 | 0.007316 | 0.2749 |
| Cyclohexanecarboxylic acid | -200470 | 1720100 | -2.8123 | 0.007697 | 0.2749 |
| Palmitelaidic acid | -1743100 | 12156000 | -2.808 | 0.007784 | 0.2749 |
| (2Z)-2-Benzylidene-6-heptenal | -59301 | 305290 | -2.8003 | 0.007938 | 0.2749 |
| Proline | 1261100 | 8590900 | 2.7826 | 0.008307 | 0.27507 |
| N,N-Diethylethanolamine | -2041700 | 6246700 | -2.7629 | 0.008735 | 0.27507 |
| NP-012534 | -8.2E+07 | 2.64E+08 | -2.7481 | 0.00907 | 0.27507 |
| [Similar to: Dodecyl sulfate; ?Mass: 1.0036 Da] | -1.1E+07 | 33657000 | -2.7409 | 0.009238 | 0.27507 |
| 1-hexadecyl-2-[(9Z,12Z)-octadecadienoyl]-sn-glycero-3-phosphocholine | 109870 | 323610 | 2.7397 | 0.009266 | 0.27507 |
| 2-[4-(3-Hydroxypropyl)-2-methoxyphenoxy]-1,3-propanediol | -46448 | 239500 | -2.7113 | 0.009956 | 0.2794 |
| NP-006255 | -1.5E+07 | 60151000 | -2.6978 | 0.0103 | 0.2794 |
| mycinose | -135040 | 583090 | -2.6947 | 0.010381 | 0.2794 |
| Frangulin B | -506740 | 2868200 | -2.6907 | 0.010488 | 0.2794 |
| XZ1800000 | -1346900 | 7773900 | -2.674 | 0.010936 | 0.28405 |
| DL-Carnitine | 22377000 | 2.07E+08 | 2.6481 | 0.011667 | 0.29566 |
| Diethyl phthalate | -87861 | 225590 | -2.6296 | 0.012216 | 0.30221 |
| (-)-Shikimic acid | -54033 | 221420 | -2.6059 | 0.012958 | 0.31309 |
| Brassilexin | -40656 | 243200 | -2.5942 | 0.013337 | 0.31493 |
| 2-Naphthalenesulfonic acid | -104310 | 238090 | -2.5694 | 0.014174 | 0.32458 |
| PC(o-18:2(9Z,12Z)/18:2(9Z,12Z)) | 112120 | 414570 | 2.5583 | 0.014566 | 0.32458 |
| (2E)-5-methyl-2-phenylhex-2-enal | -91094 | 658430 | -2.5495 | 0.014885 | 0.32458 |
| 2-(Hydroxymethyl)-4-oxobutanoic acid | -629490 | 4073800 | -2.5465 | 0.014995 | 0.32458 |
| (-)-Lupinine | -36079 | 224620 | -2.5144 | 0.016212 | 0.34376 |
| Palmitic Acid | -1E+09 | 9.51E+09 | -2.4951 | 0.016988 | 0.35276 |
| Ceramide (d18:1/9Z-18:1) | 122290 | 325030 | 2.4835 | 0.01747 | 0.35276 |
| Acetohydroxamic acid | 16258000 | 1.62E+08 | 2.4646 | 0.018284 | 0.35276 |
| beta-D-Fructofuranosyl 4-O-dodecanoyl-alpha-D-glucopyranoside | -262130 | 2869900 | -2.4596 | 0.018502 | 0.35276 |
| Testate | -105280 | 222530 | -2.459 | 0.018531 | 0.35276 |
| Nicotinamide | 26432000 | 3.59E+08 | 2.4558 | 0.018673 | 0.35276 |
| 10-Undecenoic acid | -353860 | 2066400 | -2.4213 | 0.020276 | 0.3696 |
| Dodecanedioic acid | -353860 | 2066400 | -2.4213 | 0.020276 | 0.3696 |
| 2,4-diaziran-1-yl-6-(1-phenyl-1H-pyrrol-2-yl)-1,3,5-triazine | -2092600 | 6266200 | -2.4092 | 0.020869 | 0.3722 |
| Diethanolamine | -218240 | 2391500 | -2.4005 | 0.021302 | 0.3722 |
| Isoprene | -85413 | 317260 | -2.3967 | 0.021494 | 0.3722 |
| ALA-PRO | 117500 | 563320 | 2.3822 | 0.022244 | 0.37887 |
| 11343172 | -670160 | 3756000 | -2.3733 | 0.022713 | 0.38062 |
| 1-[(3,5-dimethylisoxazol-4-yl)sulfonyl]piperidine | -161030 | 493630 | -2.348 | 0.024106 | 0.38643 |
| 1-[(3,5-dimethylisoxazol-4-yl)sulfonyl]piperidine | -161030 | 493630 | -2.348 | 0.024106 | 0.38643 |
| 2,4-diaziran-1-yl-6-(1-phenyl-1H-pyrrol-2-yl)-1,3,5-triazine | -2124100 | 5992100 | -2.3468 | 0.024175 | 0.38643 |
| Pantothenic acid | 6786600 | 53526000 | 2.3327 | 0.024983 | 0.39329 |
| UNII:6S7S02945H | -254760 | 1877400 | -2.3222 | 0.025599 | 0.39516 |
| 5,5-Dimethylhydantoin | 3996900 | 22774000 | 2.3171 | 0.025904 | 0.39516 |
| (3Z,6E)-N-Hydroxy-2,4,4,7-tetramethyl-6,8-nonadien-3-imine | -55227 | 163690 | -2.3115 | 0.026242 | 0.39516 |
| 2-(2-Ethoxyethoxy)ethanol | -47360 | 378840 | -2.3041 | 0.026696 | 0.39624 |
| 2145 | -192060 | 1322200 | -2.2961 | 0.027196 | 0.39798 |
| Caprolactam | -1195000 | 11406000 | -2.2863 | 0.02782 | 0.3982 |
| D-(-)-Glutamine | 8199400 | 48838000 | 2.2838 | 0.027977 | 0.3982 |
| 1,4,5,6-tetrahydro-6-oxonicotinic acid | -226580 | 1318600 | -2.2657 | 0.029167 | 0.40368 |
| Dibutyl malate | -145840 | 542610 | -2.2603 | 0.02953 | 0.40368 |
| MFCD00037215 | 3227600 | 12568000 | 2.2587 | 0.029637 | 0.40368 |
| Boldenone Undecylenate | -446250 | 3038500 | -2.2525 | 0.030064 | 0.40368 |
| Bis(methylbenzylidene)sorbitol | -1838800 | 12975000 | -2.2453 | 0.030559 | 0.40368 |
| Metoprolol | -4237100 | 35175000 | -2.2384 | 0.031046 | 0.40368 |
| D-(-)-Glutamine | 79602000 | 4.51E+08 | 2.2379 | 0.031082 | 0.40368 |
| L-Glutamine | 2.43E+08 | 1.14E+09 | 2.22 | 0.032371 | 0.4115 |
| Decarbamoyl-neosaxitoxin | -225020 | 616170 | -2.2186 | 0.032476 | 0.4115 |
| 2S-Amino-tridecanoic acid | -54848 | 507330 | -2.2094 | 0.033156 | 0.41505 |
| dopamine | -42320 | 365810 | -2.1991 | 0.033942 | 0.41983 |
| N,N-Diethyldodecanamide | -2537800 | 17577000 | -2.1809 | 0.035357 | 0.42662 |
| cuscohygrine | -177850 | 612640 | -2.1765 | 0.035714 | 0.42662 |
| N1-[5-(tert-Butyl)-1,3-oxathiol-2-yliden]-2,4,5-trimethylaniline | -705150 | 3269800 | -2.1764 | 0.035723 | 0.42662 |
| NP-019483 | -1463000 | 3374500 | -2.1502 | 0.037871 | 0.44264 |
| Capryloylglycine | -528780 | 4254500 | -2.1497 | 0.037916 | 0.44264 |
| trans-Anethole | -157910 | 1395000 | -2.1328 | 0.039364 | 0.44808 |
| Hydroxyamphetamine | -319210 | 2438500 | -2.1211 | 0.040399 | 0.44808 |
| Cholesterol sulfate | -1282800 | 10089000 | -2.1084 | 0.041549 | 0.44808 |
| MFCD00037215 | 1053500 | 4171700 | 2.1006 | 0.042271 | 0.44808 |
| 3-formylsalicylic acid | -194600 | 1271400 | -2.0981 | 0.042502 | 0.44808 |
| 3-Palmitolactone | -218360 | 841510 | -2.0977 | 0.042541 | 0.44808 |
| Diaveridine | -1690100 | 5229900 | -2.086 | 0.04365 | 0.44808 |
| 1H-Indole, 3-(3,7,11,15-tetramethylhexadeca-2,6,10,14-tetraenyl)- | 356240 | 610100 | 2.0824 | 0.043997 | 0.44808 |
| (2,7-Dimethyloctahydro-1H-cyclopenta[c]pyridin-4-yl)methanol | -162470 | 620330 | -2.0761 | 0.044603 | 0.44808 |
| DL-Glutamine | 211580 | 346040 | 2.0757 | 0.044644 | 0.44808 |
| (+/-)-Malic Acid | 3094000 | 18743000 | 2.072 | 0.045005 | 0.44808 |
| Hydantoin | -44261 | 416830 | -2.0709 | 0.045111 | 0.44808 |
| Diethyl sulfate | -58065 | 606920 | -2.067 | 0.045495 | 0.44808 |
| L-Pyroglutamic acid | 42521000 | 2.37E+08 | 2.0666 | 0.045537 | 0.44808 |
| L-Glutaminyl-L-proline | 48933 | 189420 | 2.0624 | 0.045961 | 0.44808 |
| UNII:74D1OHC38H | -287060 | 1899500 | -2.0596 | 0.046239 | 0.44808 |
| 3082 | -1608200 | 12023000 | -2.0583 | 0.046372 | 0.44808 |
| Lauramide | -4604700 | 50239000 | -2.057 | 0.046504 | 0.44808 |
| afegostat | 425030 | 5099500 | 2.0533 | 0.046878 | 0.44808 |
| Glu-Gly | 95717 | 1043800 | 2.052 | 0.047007 | 0.44808 |
| N-Acetyl-L-phenylalanine | 215980 | 392220 | 2.0469 | 0.047533 | 0.44897 |
| Coumarone | -171850 | 1343600 | -2.0387 | 0.04838 | 0.4493 |
| Aceglutamide | 1915100 | 3681300 | 2.0359 | 0.048672 | 0.4493 |
| 2-Butyl-5-ethyl-4-methyloxazole | -4099700 | 36246000 | -2.0341 | 0.048865 | 0.4493 |


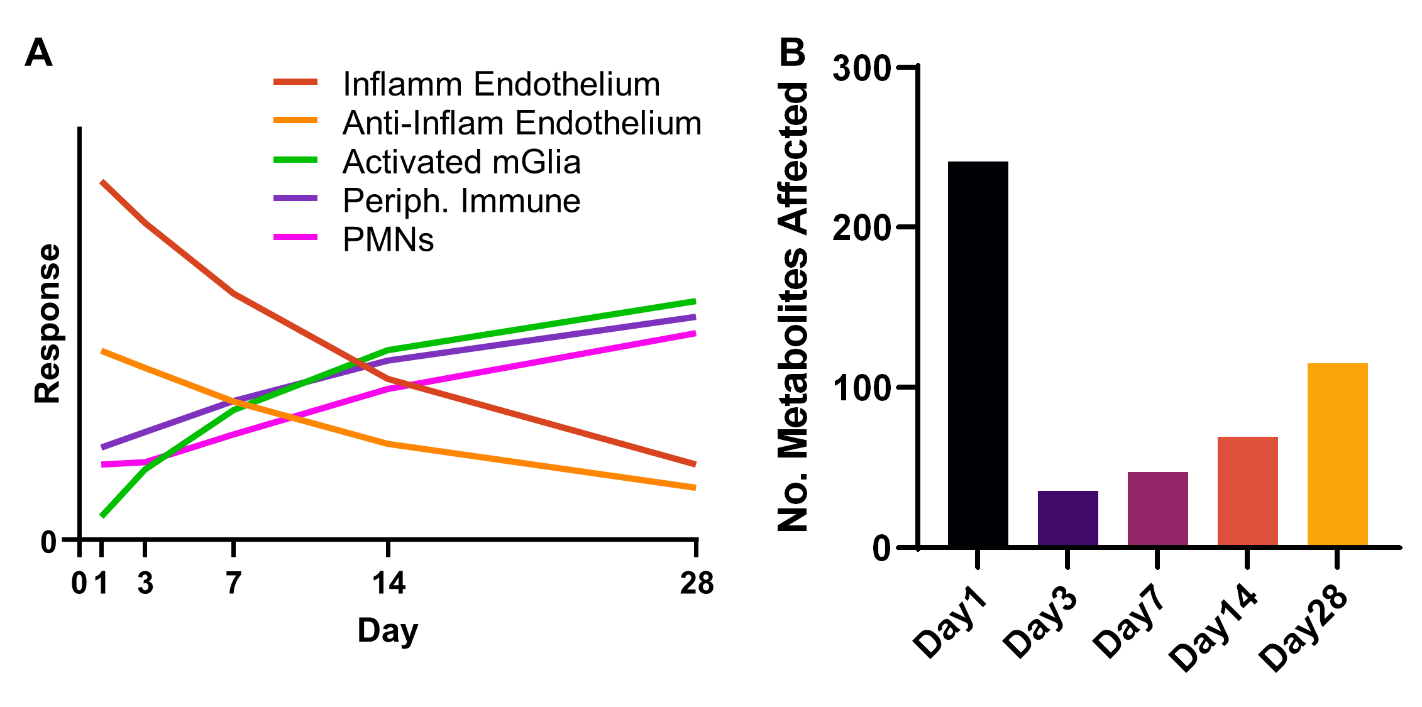


Additional file 1: **Figure S1. Neuroinflammatory and Metabolomic Temporal Dynamics.** (A) Flow cytometry significant markers were assigned a weight (population = 2, all else =1) while trending markers received half-weights (population = 1, all else = 0.5). The curves were smoothed in GraphPad and plotted (number of neighbor size = 4; 2^nd^ order polynomial). (B) Metabolites from untargeted panel Venn Diagram were counted and plotted.
